# The Occurrence and Health Risk Assessment of Aflatoxin M1 in Raw Cow Milk Collected from Tunisia during a Hot Lactating Season

**DOI:** 10.3390/toxins15090518

**Published:** 2023-08-24

**Authors:** Khouloud Ben Hassouna, Jalila Ben Salah-Abbès, Kamel Chaieb, Samir Abbès, Emilia Ferrer, Francisco J. Martí-Quijal, Noelia Pallarés, Houda Berrada

**Affiliations:** 1 Laboratory of Genetic, Biodiversity and Bio-Resources Valorisation, University of Monastir, Monastir 5000, Tunisia; khouloudhsn20@gmail.com (K.B.H.); jalila.bensalah@yahoo.fr (J.B.S.-A.); abb_samir@yahoo.fr (S.A.); 2 Laboratory of Analysis, Treatment and Valorization of Environmental Pollutants and Products, Faculty of Pharmacy, Monastir University, Monastir 5000, Tunisia; 3 Department of Biochemistry, Faculty of Science, King Abdulaziz University, Jeddah 21589, Saudi Arabia; chaieb_mo@yahoo.fr; 4 High Institute of Biotechnology of Béja, University of Jendouba, Jendouba 8189, Tunisia; 5 Nutrition and Food Science Area, Preventive Medicine and Public Health, Food Science, Toxicology and Forensic Medicine Department, Faculty of Pharmacy, Universitat de València, Burjassot, 46100 València, Spain; emilia.ferrer@uv.es (E.F.); francisco.j.marti@uv.es (F.J.M.-Q.); houda.berrada@uv.es (H.B.)

**Keywords:** aflatoxin M1, raw cow milk samples, Tunisia, HPLC-FD, risk assessment

## Abstract

Milk is a staple food that is essential for human nutrition because of its high nutrient content and health benefits. However, it is susceptible to being contaminated by Aflatoxin M1 (AFM1), which is a toxic metabolite of Aflatoxin B1 (AFB1) presented in cow feeds. This research investigated AFM1 in Tunisian raw cow milk samples. A total of 122 samples were collected at random from two different regions in 2022 (Beja and Mahdia). AFM1 was extracted from milk using the QuEChERS method, and contamination amounts were determined using liquid chromatography (HPLC) coupled with fluorescence detection (FD). Good recoveries were shown with intra-day and inter-day precisions of 97 and 103%, respectively, and detection and quantification levels of 0.003 and 0.01 µg/L, respectively. AFM1 was found in 97.54% of the samples, with amounts varying from values below the LOQ to 197.37 µg/L. Lower AFM1 was observed in Mahdia (mean: 39.37 µg/L), respectively. In positive samples, all AFM1 concentrations exceeded the EU maximum permitted level (0.050 µg/L) for AFM1 in milk. In Tunisia, a maximum permitted level for AFM1 in milk and milk products has not been established. The risk assessment of AFM1 was also determined. Briefly, the estimated intake amount of AFM1 by Tunisian adults through raw cow milk consumption was 0.032 µg/kg body weight/day. The Margin of Exposure (MOE) values obtained were lower than 10,000. According to the findings, controls as well as the establishment of regulations for AFM1 in milk are required in Tunisia.

## 1. Introduction

Milk is essential for human nutrition due to its high nutrient content and health benefits [1]. Humans consume milk either raw (unprocessed) or processed (condensed, pasteurized, powdered, liquid, heat-treated, or UHT-treated) [2]. Cow milk is the most frequently consumed by humans. When comparing cow milk and other animal milk types to human milk, it was previously thought to be the ideal food for children, who represent the majority of consumers [3].

In fact, cow milk accounts for 83% of world milk production, followed by buffalo milk with 13%, goat milk with 2%, sheep milk with 1%, and camel milk with 0.3% [4]. Furthermore, it accounts for 77% of African animal milk production [4]. The dairy sector is an important component of Tunisia’s agricultural and agro-alimentary sectors. Furthermore, in Tunisia, raw cow milk is used for making special and traditional products such as cheese and gouta. Tunisia produced an average of 1,100,000 tons of fresh cow milk per year in the period of 2010–2014 [5]. Later, in 2017, Tunisia produced approximately 1424 million liters of cow milk [6]. Its consumption by the Tunisian population is about 110 L/cap/year. This consumption is higher than the global average (100 L per capita per year) and the African average (17 L/cap/year). The cow milk collected represents more than 62.6% of the national milk produced, and the Tunisian industry dairy obtain about 89.6% of the fresh milk from collection centers [6]. Tunisia, in North Africa, is surrounded by the Mediterranean Sea, which has a warm climate with high relative humidity. These conditions are favorable to the proliferation of toxigenic molds and mycotoxins production. In fact, it is essential that milk be free of toxic compounds that can be harmful to humans, particularly children, who are more susceptible to the action of toxic compounds. In fact, the quality of milk, in terms of toxic contaminants, has a direct relation with the type and quality of animal feed. Nonetheless, milk can be indirectly contaminated by mycotoxins such as aflatoxins (AFs) and ochratoxin A (OTA) at relatively high levels, as well as fumonisins (FBs), zearalenone (ZEA), and deoxynivalenol (DON) at trace levels, by ingesting contaminated feed [7]. Mycotoxins are a group of highly toxic secondary metabolites produced by *Aspergillus*, *Fusarium*, *Penicillium*, *Claviceps*, and *Alternaria* fungal species [8]. Mycotoxins can cause health risks ranging from allergic reactions to death, both in humans and animals. In effect, their toxicity is determined by the concentration and type of mycotoxins consumed. They are responsible for chronic effects, namely immunotoxicity, genotoxicity, hepatotoxicity, nephrotoxicity, teratogenicity, cytotoxicity, and neurotoxicity [9]. Furthermore, the International Agency for Research on Cancer has classified AFs as carcinogenic to humans and animals [10].

The majority of studies on mycotoxins’ presence in milk have focused on aflatoxin M1 (AFM1) analysis. AFM1 is a hepatic carcinogenic mycotoxin, and its presence in milk poses a danger for humans [11]. In fact, AFM1 is a monohydroxilated product of aflatoxin B1 (AFB1) biotransformation in the cow liver, and is detected in milk within 12–24 h of the first ingestion of AFB1 presented in animal feed [12]. It is excreted in milk at rates ranging from 1% to 6% of the dietary consumption [13]. The rate of biotransformation varies between animals, and other factors such as diet, rate of ingestion, digestion rate, animal health, and the biotransformation capacity of the liver [14]. AFM1 was classified as a Group 1 carcinogen by the International Agency for Research on Cancer [10]. Milk contamination by mycotoxins is not only a public health problem, but it also costs farmers financially due to the negative effects of them on animal production [15]. The presence of AFM1 in milk is a worldwide issue. Once it is secreted into milk, it cannot be removed by technological treatments such as pasteurization, sterilization, or drying [16]. In order to protect humans, the European Union established 0.05 µg/L as the maximum permitted level (MPL) of AFM1 in milk [17]. Similarly, the Food and Drug Administration (FDA) in the United States has established a maximum permitted level of 0.50 µg/L for AFM1 in milk [18]. Tunisia, unfortunately, does not have a maximum limit for mycotoxins in milk and milk products.

For the detection of mycotoxins in milk, many sensitive, selective, and reliable analytical techniques are used, such as enzyme-linked immunosorbent assay (ELISA) and liquid chromatography coupled with a fluorescence or ultraviolet detector (HPLC-FD/HPLC-UV), thin-layer chromatography (TLC), LC-MS/MS, and ultra-performance liquid chromatography coupled with tandem mass spectrometry (UHPLC-MS/MS) [19,20,21].

Despite the high consumption of cow milk by Tunisian people and the dangers that mycotoxins pose to humans and animals, to the best of our knowledge, only one study about mycotoxins presence in milk was carried out in Tunisia [22], and no data on the risk assessment of mycotoxin consumption was available in this study. Thus, it is essential to investigate the occurrence of mycotoxins in milk to furnish information about human exposure. Taking those considerations into account, the objectives of the present study are to determine and quantify AFM1 in raw cow milk collected from two Tunisian regions, Beja (the continental region) and Mahdia (the coastal region), using HPLC-FD, as well as to determine the daily exposure of Tunisians consumers to AFM1 through cow milk consumption.

## 2. Results

### 2.1. AFM1 Levels in Raw Cow Milk

One hundred and twenty-two samples of raw cow milk produced in the Tunisian provinces of Mahdia (n = 60) and Beja (n = 62) were analyzed by HPLC-FD to evaluate the occurrence of AFM1. The AFM1 occurrence, concentration range, and mean concentration are presented in Table 1. In fact, only positive samples above the LOD (0.003 µg/L) were considered.

This study exposed high contamination levels of AFM1 in the raw cow milk collected in Tunisia. AFM1 was found in 119 out of 122 (97.54%) of analyzed milk samples, at concentrations ranging between values lower than the LOQ and 197.37 µg/L, with a mean value of 50.44 µg/L. However, it is important to highlight that only 18 of 119 positive samples were contaminated at levels above LOQ. Furthermore, AFM1 was not detected in 3 of 122 samples. Moreover, 18 of 122 analyzed samples exceeded the maximum permitted level (MPL) set for AFM1 by the EU (0.05µg/L) [17] and the FDA 0.5 µg/L [18].

### 2.2. Regional Differences in AFM1 Contamination Levels

Based on the milk sampling region, the levels of AFM1 in samples collected from two different regions in Tunisia were compared (Table 2 and Table 3). The highest AFM1 maximum concentration was found in the continental region (Beja), at 197.37 µg/L, compared to 99.36 µg/L in the coastal region (Mahdia). Moreover, the mean value of AFM1 in milk samples from the continental region (64.27 µg/L) was about 1.6 times higher than in milk samples from the littoral region (39.73 µg/L). AFM1 levels of some samples, eight from the Beja region and ten from Mahdia, were above the thresholds set by the EU and FDA [17,18].

### 2.3. Estimation of Daily Intake

Based on the findings of this study and the amount of milk consumed by the Tunisian population (approximately 110 L/person/year [23]), the estimated daily intake of AFM1 (EDI) for a Tunisian adult weighing 70 kg was estimated to be 0.032 µg/kg body weight/day, considering the mean concentration calculated under Lower Bound scenario (LB) and Upper Bound scenario (UB). 

### 2.4. AFM1 Risk Characterization

The characterization of AFM1 risk from dairy product consumption in the Tunisian population was performed using a deterministic approach. The Margin of Exposure (MOE) was calculated. Considering the LB and UB scenarios the MOE value for raw milk was 12.54.

## 3. Discussion

### 3.1. AFM1 Presence in Raw Cow Milk

Mycotoxins are a worldwide issue that harms both animals and humans. Similar to the rest of the world, the Tunisian people consume a great quantity of cow milk. The determination of AFM1 (a carcinogenic mycotoxin) levels in raw cow milk samples from Tunisia, as well as the investigation of the risk assessment associated with this contamination, piqued our interest in this study. In the context of this study, there are other studies on AFM1 occurrence in raw cow milk from countries over the world highlighting their presence. To the extent of our knowledge, only one study on mycotoxins in milk has been published in Tunisia [22].

In this survey, AFM1 contamination levels in raw milk were found in 97.54 percent of analyzed samples. Similar to our results, AFM1 was found in raw cow milk samples with very high occurrences in various other North African countries. In Egypt, Kamel et al. [24] used UPLC-MS/MS to analyze raw cow milk samples and found AFM1 in all of them (100%) at quantities ranging from 0.14 to 8.54 µg/L. Later, Abdallah et al. [25] found AFM1 in 20 of 20 samples taken from different local markets in Assiut Governorate in Upper Egypt, with concentrations ranging from 0.02 to 0.19 µg/L. In a previous Sudanese investigation, Ali et al. [26] also revealed AFM1 in all raw milk samples tested with concentrations ranging from 0.1 to 2.52 µg/L. In 2023, Hamed et al. [27] used HPLC-FD to analyze raw cow milk from Egypt, and detected AFM1 in 88% of samples with a maximum concentration of 0.122. 

More recently, in 2023, AFM1 was detected in all of the raw cow milk samples from Ethiopia [28]. However, no AFM1 occurrence was detected in raw cow milk from Portugal in 2023 [29]. North African counties, including Tunisia, which are surrounded by the Atlantic Ocean, the Mediterranean Sea, and the Red Sea, have a humid and warm climate that may encourage fungal development and mycotoxin production. In fact, the high temperatures associated with climate change induce fungi apparition and mycotoxin production in animal feeds [30]. Recently, The Food and Agriculture Organization (FAO) [31] have published a paper on climate change and food safety, notably analyzing the effects of climate change on mycotoxins. In the sampling period, the temperature (21 °C) was high (Table 2). Aflatoxins (AFs), including AFB1, are mycotoxins produced in animal feed by fungal genera such as *Aspergillus flavus*, *Aspergillus parasiticus*, and *Aspergillus nomius* [32]. These fungi can grow at temperatures ranging from 19 to 35 °C [33], producing AFB1 in Tunisian animal feed [34]. In comparison to our findings, AFM1 contamination has been recorded in other studies with zero or low prevalence in raw cow milk samples. In North Africa, lower incidences were reported. In 2015, Redouane et al. [35] reported that an only one out of 22 (4.5%) raw cow milk sample from Algeria was contaminated by AFM1. Also in Egypt, a study revealed AFM1 in only 18% of raw cow milk samples taken from Cairo provinces [36], and later Zakaria et al. [37] found AFM1 in 49% of raw milk from the Aswan province. In an Algerian study, the contamination of raw cow milk was shown to have an incidence of 46.43% [38].

The concentrations found in this work ranged from values below LOQ and 197.37 µg/L. In accordance with our results, Esam et al. [39] found high concentrations of AFM1, ranging between 10 and 110 µg/L in all raw cow milk samples analyzed from Egypt. Added to that, Mukhtar et al. [40] detected the presence of AFM1 in the overall fresh raw cow milk samples collected from Nigeria at concentrations ranging from 22.33 to 119.99 µg/L.

The high AFM1 concentration in milk is usually explained by the contamination of cow feeds by AFB1. According to Table 2, the sampling period was a dry one during which farmers were not able to feed their animals (kept in local dairy farms) with green feed. Therefore, the main feed supplements are grains (corn and soya), and concentrated feed is a mixed of barley, wheat, corn, and soya, which are susceptible to mycotoxins and fungal contamination due to poor transportation and storage conditions. Tunisians typically store those feeds in non-hermetic bags made by jute and woven polypropylene. These bags have inadequate construction because they allow air to enter, exposing the stored feed to fungus spores [41].

The maximum concentration of AFM1 (197.37 µg/L) in Tunisian raw milk was higher than that reported in other countries around the world, including Cyprus (0.017 µg/L; [42]), Turkey (0.034 µg/L; [43]), Italy (0.026 µg/kg; [44]), Pakistan (0.021 µg/L; [45]), Brazil (0.045 µg/L; [46]), China (0.036 µg/L; [47]), Lebanon (0.440 µg/L; [48]), and Spain (1.36 µg/L; [49]), and lower than that reported in Kenya (273.8 µg/L; [50]) and Iran (240 µg/L; [51]).

Those different results indicate that milk contamination levels with AFM1 vary between countries. These differences are due to different techniques for extracting and detecting mycotoxins, the type and quality of forage and feed, the kind of cow diet, the geographic location, the climatic variation, the sampling seasons, the genetic variation in dairy cows, the farming systems, and the feed storage [51,52,53].

In terms of regulations, all raw milk samples had AFM1 concentrations that exceeded the MPL (0.050 µg/L for the EU and 0.5 µg/L for the FDA). In the same way, the percentage of AFM1 contamination levels of samples exceeding regulatory limits have been reported for cow milk from north Africa, such as Egypt (100%; [24]) and Sudan (100%; [26]); however, Mohammedi-Ameur et al. [38] found that only one sample (1.19%) of Algerian raw milk had an AFM1 concentration above the MPL. Cow milk contamination levels (%) that exceeded regulatory limits have also been reported in other African countries such as Burundi (100%; [54]), Kenya (63%; [55]), Zimbabwe (79.2%; [56]), and Tanzania (83.8%; [57]), as well as in other countries over the world such as Iran (83.7%; [58]) and Indonesia (68.4%; [59]).The high quantities of AFM1 reported in this study can be attributed to the nature of the milk examined, which was fresh and untreated. In a Moroccan study, 15.2% of pasteurized milk samples were contaminated with AFM1 at lower levels ranging from 0.010 to 0.077 µg/L while in ultra-high temperature treated (UHT) milk, only 9.5% of samples were contaminated with AFM1 at concentrations ranging from 0.013 to 0.048 µg/L [60]. AFM1 was found in three different milk products from Punjab, India, with varying levels of contamination (percentage of positive samples/maximum concentration), including raw milk (80%, 4.185 µg/L), pasteurized milk (41%, 2.330 µg/L), and ultra-high-temperature milk (47%, 2.585 µg/L) [61]. Inadequate conditions, contamination of the milking instrument and/or manipulation of raw milk may also contribute to mycotoxin contamination of milk [62].

### 3.2. Regional Differences in AFM1 Concentrations

The current study reported considerable differences in AFM1 contamination levels in raw milk samples from two different regions in Tunisia, with the continental region having the highest contamination levels with a mean concentration of 64.27 µg/L and all the positive samples having concentrations above the MPL (0.05 µg/L). However, Abbès et al. [22] found contradictory findings when analyzing raw cow milk from the continental region, Beja, in Tunisia using ELISA technique. They detected moderate contamination levels of AFM1, with a mean concentration of 13.62 µg/L, and only 5 (4.4%) had concentrations above 0.05 µg/L. The differences in concentrations observed could be attributed to geographic and climatic variations [63]. In fact, high levels of AFM1 in milk samples were found in continental regions with more precipitation, including rain (Table 2), which increases humidity and favors the growth of aflatoxigenic fungi and the accumulation of aflatoxins, in particular AFB1, in dairy animal feed, which was then transferred to milk in the form of AFM1 [64]. These variations could also be caused by other factors such as animal species, milking time, the level of mycotoxins in feed intake, and the volume of milk produced by the animal mammal in such region. In fact, due to limited resources for this survey, we could not collect background information on farming practices and cow feeds (green feed, dry feed, and concentrate) provided at the different regions. So far, we have no information about differences in AFM1 contamination.

### 3.3. Estimated Daily Consumption of AFM1

Humans are exposed daily to AFM1 through the consumption of contaminated milk. Exposure assessment, as one component of risk assessment methodology, combines mycotoxin levels in food with consumption habits, providing valuable information for risk management if mycotoxins affect food safety and health on an individual or population level [65]. The estimated exposure of humans to mycotoxins, in particular AFM1, is calculated by combining contamination and consumption data. In this context and, for the first time in Tunisia, the determination of the EDI of AFM1 was carried out to evaluate the risk associated with the intake of this mycotoxin through milk consumption by Tunisian adults (70 kg). In Tunisia, milk consumption was estimated at 110 L/person/year. This consumption is compared with neighboring countries. It was higher than 72 L/person/year in Morocco and lower than 140 L/person/year in Algeria [60]. In this survey, considering the mean of total samples, EDI values obtained were 0.032 µg/kg body weight/day in both the LB and UB scenarios. The great finding of EDI can be explained by the Tunisian population’s higher intake of raw cow milk, due to its low cost, accessibility, and taste. It can also be explained by the high prevalence of AFM1 in the raw milk samples collected, given that this toxin is not removed or degraded by the industry’s typical technological processes, such as heat treatments, drying, and/or milk fermentation [60].

Although various studies have reported the presence of AFM1 in milk [66], few data on the risk assessment of this mycotoxin for milk consumers are still accessible, and no data are available in Tunisia. In comparison to data from other countries, the EDI (0.032 µg/kg body weight/day) of AFM1 taken from cow raw milk consumption reported in this research was the highest. In comparison to our reported data, negligible EDI values were reported in other studies. Bilandžić et al. [67] found EDI values from mean raw milk intake ranging from 0.000017 to 0.00282 µg/kg bw/day in a Croatian study. Further, Mohammedi-Ameur et al. [38] found an EDI of 0.0003425 µg/kg bw/day in raw milk from Algeria. Also, the research by Gizachew et al. [68] reported an EDI of 0.0007891 µg/kg bw/day in raw milk from Ethiopia. Recently, Daou et al. [48] found an EDI of 0.0001126 µg/kg bw/day for raw milk in Lebanon. In Ghana, the EDI ranged between 0.000006 and 0.000203 µg/kg bw/day for infants, toddlers, children, teenagers, and adults [69]. Due to the lack of consumption data for various age groups in this study, we were able to estimate exposure and health risks only for adults.

### 3.4. Risk Characterization of AFM1 Exposure

Regarding the MOE, it can be used to describe the health risk posed by carcinogenic and genotoxic substances found in food [70]. When the value of the MOE is ≥ than 10,000, it is assumed that there is a low risk to public health [66]. In our study, the MOE values measured in both scenarios were less than 10,000 (12.54 in both the LB and UB scenarios). In any case, the ingestion of raw cow milk contaminated with AFM1 can result in a public health problem (liver cancer). Similar to our results, Kortei et al. [69] found MOE values ranging from 197 to 6666.7, which was below 10,000 and caused a problem for public health by consuming raw cow milk. Further, Zebib et al. [28] revealed that the MOE detected in the studied Ethiopian regions was less than 10,000 in adult populations, indicating a possible public health risk due to the high AFM1 exposure from raw milk consumption. Contrary to our findings, the MOE values for Serbian consumers’ exposure to AFM1 were significantly higher than 10,000 in toddlers and other children, indicating that there is no health risk associated with AFM1 exposure through consuming milk [71]. The differences in results reported in this work and other studies could be attributed to a variety of factors, including milk consumption, nutritional habits, the number of analyzed samples, the milk sampling period, and the method used for AFM1 extraction and analysis (ELISA kits, HPLC, or LC-MS/MS, for example). That said, attention should be paid to the health risks associated with exposure to mycotoxins, including AFM1, through consumption of raw cow milk.

The high frequencies and concentrations of AFM1 in raw milk samples in the current investigation indicate that lactating cows were exposed to high levels of dietary AFB1. Thus, preventive approaches for reducing AFB1 concentrations in feed as well as good storage practices and hygiene in dairy farms should be explored to protect animal health. Furthermore, to protect consumers from possible health risks associated with AFM1 exposure, more extensive and periodic control of AFM1 concentration in milk is required, as is establishing regulations for mycotoxins in Tunisia.

## 4. Conclusions

In the current survey, AFM1 was frequently identified in raw cow milk from two regions of Tunisia (the continental and coastal regions) with high levels of contamination. The amounts of AFM1 in 18 of 119 positive samples exceeded the MRL. According to the toxicological parameters of EDI and MOE calculated in this study, where the MOE was less than 10,000, there was a public health problem, particularly hepatocellular carcinoma (HCC), due to the high exposure of the Tunisian population to AFM1 by consuming raw cow milk. To avoid the proliferation of fungi and the production of aflatoxins, farmers must be informed of the optimal storage conditions for animal feed as well as milk control. Strict regulation and a tolerable maximum for AFB1 and AFM1 should be established in Tunisia to reduce possible risks to health and economic losses. Further investigation of AFM1 in milk and its risk assessment in consumers, particularly children (the majority of milk consumers), is needed.

## 5. Materials and Methods

### 5.1. Reagents and Chemicals

The solvents used in this work, methanol (MeOH) and acetonitrile (ACN) (HPLC grade), were acquired from Merck (Darmstadt, Germany). The deionized water (resistivity > 18 M cm1) was obtained in the laboratory from a Milli-Q SP^®^ Reagent Water System (Millipore Corporation, Bedford, MA, USA). The formic acid (95%) was purchased from Sigma Aldrich (St. Louis, MO, USA). The Nylon filters (0.45-μm pore size) were supplied by Scharlau (Barcelona, Spain). The syringe nylon filters (13 mm diameter and 0.22 m pore size) were supplied by Membrane Solutions (Plano, TX, USA). The derivatization reagent (pyridine hydrobromide perbromide, tech.90%) was acquired from Thermo Scientific (Kandel, Germany). The glacial acetic acid (99%) was supplied by Fisher Scientific (Leics, UK). The hexane (95%) was provided by VWR International bvba (Leuven, Belgium). The tri-sodium citrate dihydrate was purchased from VWR International BVBA (Leuven, Belgium). The disodium hydrogen citrate sesquihydrate (99%) was delivered from Alfa Aesar GmbH and Co. KG (Karlsruhe, Germany). The sodium chloride (99.5%) was supplied by Fisher Scientific (Loughborough, UK). The magnesium sulfate anhydrous was supplied by Thermo Scientific (Kandel, Germany). The standards for Aflatoxin M1 were purchased from Sigma Aldrich (Madrid, Spain). All solutions were kept in amber vials under secure conditions at −20 °C. Prior to injection into the LC-FD system, these stock solutions were diluted to obtain adequate working concentrations.

### 5.2. Study Region and Samples Collection

A total of 122 raw cow milk samples were randomly collected from two different geographical and agro-climatic conditions regions in Tunisia: the continental region (n = 62), defined as Beja, characterized by a more arid climate, and the coastal region (n = 60), represented by the location of Mahdia, characterized by a more semi-arid climate. Table 3 shows the geographic information for the coastal and continental areas. A total of 40 mL of fresh samples were collected directly from dairy cows in farmers’ houses and dairy farms during a routine midday milking procedure. Samples were stored in 50 mL Falcon tubes at −20 °C until analysis without being treated in any way beforehand. All samples were transported to Valencia, Spain, in polystyrene cartons containing dry ice. Then, they were kept in the laboratory at −20 °C until the analysis.

### 5.3. AFM1 Extraction from Milk

A QuEChERS extraction procedure was employed to extract AFM1 from milk. To summarize, 500 µL of milk sample were placed in a 15-mL Falcon tube. Then, 2 mL of hexane were added to the sample. The mixture was vortexed for 30 s before being centrifuged for 5 min at 5000 rpm in an Eppendorf Centrifuge 5810R (Eppendorf, Hamburg, Germany). Following the removal of the hexane, 2.5 mL of deionized water and 2.5 mL of ACN were added to the tube and shaken for 1 min. Subsequently, a mix of salts (2 g of MgSO_4_, 0.3 g of NaCl, 0.25 g of DSHCSH, and 0.5 g of TSCDH) was added. After vortexing for 1 min and centrifugation for 5 min at 3500 rpm, 2 mL of the upper phase was filtered through a 13 mm/0.22 μm nylon filter. Subsequently, 900 µL of the filtrate were dried at 40 °C under a nitrogen stream using a multi-sample Turbo-vap LV Evaporator (Zymark, Hoptkinton, USA). Finally, and before HPLC analysis, the final residue was reconstituted with 300 µL of methanol.

### 5.4. AFM1 Determination by HPLC-FD

A JASCO Lc-Net II/ADC coupled to a FP-2020 plus JASCO detector was used for the determination of AFM1. The chromatographic separation was achieved with a liquid purple ODS reverse-phase column C18 (5 µm, column LC 150 × 4.6 mm, Analisis vinicos). The mobile phases consisted of acetonitrile (A), 0.1% acetic acid water (B), and methanol (C). The gradient program began with a proportion of 15% for eluent A and 60% for eluent B. Then, mobile phase A changed to 50% and 40% of mobile phase B in 14 min and remained the same proportion until 30 min. Then, the column was cleaned and readjusted to initial conditions over the next 5 min. The following instrument parameters were set: injection volume of 10 µL, flow rate of 0.8 mL/min, excitation wavelength of 365 nm, and emission wavelength of 455 nm.

The methodology proposed for AFM1 determination was optimized in terms of recoveries, matrix effects, linearity, and limits of detection (LOD), and quantification (LOQ). All analytical parameters obtained were in accordance with the criteria established by European Commission Decision 2002/657/EC. In this sense, good recoveries were observed at 100 μg/L, with intra-day and inter-day precision between 97 and 103%, respectively. SSE (%) obtained evidenced no matrix effects. Calibrations curves constructed by spiking blank raw milk extract samples and solvent at levels comprised between <LOQ and 250 μg/L revealed good linearity, with correlation coefficients (R^2^) between 0.990 and 0.999. Finally, the LOD and LOQ were 0.003 μg/L and 0.01 μg/L, respectively. Matrix-matched calibration curves were used for effective quantification of milk samples.

### 5.5. Estimation of the AFM1 Daily Intake (EDI)

The EDI was determined by the AFM1 mean concentration found in analyzed milk samples, the daily intake of milk by the population, and the average body weight of an adult consumer. The EDI was calculated using the following formula [72] and expressed in µg/kg per body weight (bw) per day [66].

$$EDI=\frac{CAFM1\times ADC}{BW}$$

where CAFM1 is the mean AFM1 concentration in milk samples (µg/L), ADC is the average daily consumption of milk (L/day), and BW is the body weight of an individual consumer (kg).

To consider those concentration values that were below the LOD or LOQ (left-censored data), an exposure assessment was performed in two different scenarios [73,74]: the lower bound scenario (LB), in which the value of 0 was attributed when AFM1 was not detected or detected below the LOQ, and the Upper bound scenario (UB), in which the value of LOD was attributed to AFM1 that was not detected, and the value of LOQ was attributed to AFM1 detected at levels below the LOQ.

### 5.6. Risk Characterization Margin of Exposure (MOE)

The MOE was determined by dividing the Benchmark dose lower limit of 10% (BMDL 10) for AM1, which was 0.4 µg/kg bw/day (AFM1 potency in male Fischer rats based on a 2-year study) by EDI [66]. The benchmark is an estimate of the lowest dose that is 95% certain to cause no more than 10% cancer incidence; it is the dose that produces a minor but discernible reaction [28].

The main finding of the risk assessment of aflatoxins, including aflatoxin M1, was liver carcinogenicity. In fact, a MOE value below 10,000 indicates that exposure to these mycotoxins may increase the risk of developing hepatocellular carcinoma (HCC), which is a serious public health concern [66].

### 5.7. Statistical Study

The differences between AFM1 occurrence levels of the samples were evaluated using a randomized block experiment. Furthermore, significance level between sampling periods was elucidated using Duncan multiple comparison test. A multi sample ANOVA test was used also for determination of the significance (*p* value < 0.05).

## Figures and Tables

**Table 1:** Incidence of the studied AFM1 (μg/L) in raw cow milk samples from tow Tunisian regions.

Region	Positive Samples		<LOD	<LOQ	Minimum Concentration (µg/L)	Maximum Concentration (µg/L)	Mean Concentration (µg/L)	Lower Bound Scenario (µg/L)	Upper Bound Scenario (µg/L)
	n	%							
Mahdia (60)	58	96.67	2	48	<LOQ	99.36	39.37	6.56	6.57
Beja (62)	61	98.39	1	53	<LOQ	197.37	64.27	8.29	8.30
Total (122)	119	97.54	3	101	<LOQ	197.37	50.44	7.44	7.45

Only positive samples (≥LOD: 0.003 µg/L) were considered for calculations. Lower bound scenario (LB): the value of 0 was attributed when AFM1 was not detected or detected below the LOQ. Upper bound scenario (UB): the value of LOD was attributed to AFM1 that was not detected, and the value of LOQ was attributed to AFM1 detected at levels below the LOQ. Values are expressed as means of three assays and significant vs. control assay: *p* < 0.05. LOD: limit of detection: 0.003 µg/L. LOQ: limit of quantification: 0.01µg/L.

**Table 2:** Meteorological data during sampling period of 2022 in Tunisia.

Region (Period)	Average of Precipitation (mm)	Average of Temperatures (°C)	Average of Humidity (%)
Littoral region (March–June)	3.75	21.25	71.5
Continental region (March–June)	10.5	21.25	71

Data from the National Institute of Meteorology (Tunisia).

**Table 3:** Geographic data related to the sampling regions in Tunisia.

Sampling Region	Geographic Position	Altitude (m)	Latitude	Longitude	Bioclimatic Zone
Mahdia (Coastal)	Center East	6	36807 N	10_22051 E	Semi-Arid Inferior
Beja (Continental north)	Northwest	93	36_33004 N	9_26035 W	Sub-Humid

## Data Availability

Not applicable.
